# Early-Stage Alzheimer's Disease Prediction Using Machine Learning Models

**DOI:** 10.3389/fpubh.2022.853294

**Published:** 2022-03-03

**Authors:** C. Kavitha, Vinodhini Mani, S. R. Srividhya, Osamah Ibrahim Khalaf, Carlos Andrés Tavera Romero

**Affiliations:** ^1^Department of Computer Science and Engineering, Sathyabama Institute of Science and Technology, Chennai, India; ^2^Al-Nahrain Nanorenewable Energy Research Center, Al-Nahrain University, Baghdad, Iraq; ^3^COMBA R&D Laboratory, Faculty of Engineering, Universidad Santiago de Cali, Cali, Colombia

**Keywords:** healthcare, prediction, Alzheimer's disease (AD), machine learning, feature selection

## Abstract

Alzheimer's disease (AD) is the leading cause of dementia in older adults. There is currently a lot of interest in applying machine learning to find out metabolic diseases like Alzheimer's and Diabetes that affect a large population of people around the world. Their incidence rates are increasing at an alarming rate every year. In Alzheimer's disease, the brain is affected by neurodegenerative changes. As our aging population increases, more and more individuals, their families, and healthcare will experience diseases that affect memory and functioning. These effects will be profound on the social, financial, and economic fronts. In its early stages, Alzheimer's disease is hard to predict. A treatment given at an early stage of AD is more effective, and it causes fewer minor damage than a treatment done at a later stage. Several techniques such as Decision Tree, Random Forest, Support Vector Machine, Gradient Boosting, and Voting classifiers have been employed to identify the best parameters for Alzheimer's disease prediction. Predictions of Alzheimer's disease are based on Open Access Series of Imaging Studies (OASIS) data, and performance is measured with parameters like Precision, Recall, Accuracy, and F1-score for ML models. The proposed classification scheme can be used by clinicians to make diagnoses of these diseases. It is highly beneficial to lower annual mortality rates of Alzheimer's disease in early diagnosis with these ML algorithms. The proposed work shows better results with the best validation average accuracy of 83% on the test data of AD. This test accuracy score is significantly higher in comparison with existing works.

## Introduction

Alzheimer's Disease (AD) is a progressive neurological condition that leads to short-term memory loss, paranoia, and delusional ideas that are mistaken for the effects of stress or aging. In the United States, this Disease affects about 5.1 million people. AD does not have proper medical treatment. In order to control AD, continuous medication is necessary. AD (1) is chronic so that it can last for years or the rest of your life. Therefore, it is most important to prescribe medication at the appropriate stage so that the brain is not damaged to a great extent. Early detection of this Disease is a tedious and costly process since we must collect a lot of data and use sophisticated tools for prediction and have an experienced doctor involved. Automated systems are more accurate than human assessment and can be used in medical decision support systems because they are not subject to human errors. Based on previous research on AD, researchers have applied images (MRI scans), biomarkers (chemicals, blood flow), and numerical data extracted from the MRI scans to study this Disease. As such, they were able to determine whether a person was demented or not. In addition to shortening diagnosis time, more human interaction will be reduced by automating Alzheimer's diagnosis. In addition, automation reduces overall costs and provides more accurate results. For example, we can predict whether a patient is demented by analyzing MRI scans and applying prediction techniques. If a person has early-stage Alzheimer's Disease, they are considered demented. By doing so, we can achieve better accuracy.

When a person has Alzheimer's Disease in the early stages, they can usually function without any assistance. In some cases, the person can still work, drive, and partake in social activities. Although this is the case, the person may still feel uneasy or suffer from memory loss, such as not remembering familiar words and locations. People close to the individual notice that they have difficulty remembering their names. By conducting a detailed medical interview, a doctor may identify problems with memory and concentration in the patient. Common challenges in early stage of Alzheimer's Disease include,

- It's hard to remember the right word or name.
- Having difficulty remembering names when meeting new people.
- Working in social settings or the workplace every day can be challenging.
- Having forgotten something that you have just read in a book or something else.
- Having trouble finding or misplacing a valuable object.
- Tasks and activities are becoming increasingly difficult to plan or organize.

Alzheimer's symptoms become more persistent as the Disease progresses. When people suffer from dementia, their ability to communicate, adapt to their environment, and eventually move is lost. It becomes much more difficult for them to communicate pain through words or phrases. Individuals may need substantial assistance with daily activities as their memory, and cognitive skills continue to decline. At this stage, individuals may:

- Personal care and daily activities require 24/7 assistance.
- The consciousness of their surroundings, as well as recent experiences, is lost.
- As you age, you may experience changes in your physical abilities and walking, sitting, and eventually swallowing.
- Communication with others is becoming increasingly difficult.
- Infections, specifically pneumonia, become more prevalent.

### Motivation

Under the current conditions, human instinct and standard measurements do not often coincide. In order to solve this problem, we need to leverage innovative approaches such as machine learning, which are computationally intensive and non-traditional. Machine learning techniques are increasingly being used in disease prediction and visualization to offer prescient and customized prescriptions. In addition to improving patients' quality of life, this drift aids physicians in making treatment decisions and health economists in making their analyses. Viewing medical reports may lead radiologists to miss other disease conditions. As a result, it only considers a few causes and conditions. The goal here is to identify the knowledge gaps and potential opportunities associated with ML frameworks and EHR derived data.

### Contribution

In our research work, people affected by Alzheimer's Disease are identified and we aims at finding individuals who potentially have Alzheimer's at an early stage. The datasets for Alzheimer's Disease is available on both OASIS and Kaggle which is used for training all patient's data using various machine learning algorithms such as SVM, Random Forest classifier, Decision tree classifier, XGBoost and Voting classifier to effectively distinguish the affected individuals with high degree of efficiency and speed. Finally, an overview of how the Disease has affected the population according to various criteria is analyzed.

### Organization

Following are the different sections of our work: Section Related Works address the recent papers on detecting Alzheimer's Disease using Machine learning and Deep learning models. Section Materials and Methods discusses the exploratory data analysis, and different Machine learning classifier models. Results and Discussion section address the performance measures of different Machine Learning models. Finally Section Conclusion concludes the work and discusses the future work.

## Related Works

Alzheimer's Disease is predicted using ML algorithms by using a feature selection and extraction technique, and the classification is conducted based on the oasis longitudinal dataset. The different techniques (2) involved in analyzing brain images for diagnosing diseases of the brain to provide a brief overview. Several major issues are discussed in this article relating to machine learning and deep learning-based brain disease diagnostics based on the results of the reviewed articles. The most accurate method of detecting brain disorders was found in this study and can be used to improve future techniques. Using machine learning and deep learning platforms, this study aims to combine recent research on four brain diseases: Alzheimer's disease, brain tumors, epilepsy, and Parkinson's disease. By using 22 brain disease databases that are used most during the reviews, the authors can determine the most accurate diagnostic method.

Martinez-Murcia et al. (3) uses deep convolutional autoencoders to explore data analysis of AD. Data-driven decomposition of MRI images allows us to extract MRI features that represent an individual's cognitive symptoms as well as the underlying neurodegeneration process. A regression and classification analysis are then performed to examine the distribution of the extracted features in a wide variety of combinations, and the influence of each coordinate of the autoencoder manifold on the brain is calculated. MMSE or ADAS11 scores, along with imaging-derived markers, can be used for over 80% accuracy to predict AD diagnosis.

A deep neural network is used with layers (4, 5)) that are all connected to perform binary classification. Each hidden layer is activated by a different activation function. A model with the best performance is chosen after k-folds validation Researchers at the Lancet Commission found that about 35% of Alzheimer's risk factors can be modified. The following factors can contribute to these risks: a lack of education, hypertension, obesity, hearing loss, depression, diabetes, lack of physical activity, smoking, and social isolation. Regardless of the impact of these factors at any stage of life, it is beneficial to eliminate them. Studies have suggested (6) that early intervention and treatment of modifiable Alzheimer's risk factors can prevent or delay 30% of cases of Alzheimer's (7). According to the Innovative Midlife Intervention for Alzheimer's Deterrence (In-MINDD) project (8) one way to calculate Alzheimer's risk based on risk factors is by using the Lifestyle for Brain Health (LIBRA) index (9–12). According to the National Academy of Medicine (13, 14) cognitive training, hypertension management, and increased physical activity were the three main categories of dementia intervention. The most common type of Alzheimer's is Alzheimer's Disease (AD). Among the types of Alzheimer's, Vascular Alzheimer's (VaD) is the second most common, followed by Alzheimer's with Lewy bodies. A few other types of Alzheimer's are associated with brain injuries, infections, and alcohol abuse. Tatiq and Barber (15) in their study suggested that Alzheimer's can be prevented by targeting modifiable vascular risk factors because these two types often co-exist in the brain and share some modifiable risk factors. Williams et al. (16) obtained predictions of cognitive functioning based on neuropsychological and demographic data using four different models: SVM, Decision Tree, NN, and Naïve-Bayes. In this case, average values were substituted for the missing values; the accuracy of Naive Bayes was the highest. Data from ADNI study are applied using ten-fold cross-validation (17, 18) and show high correlation between genetic, imaging, biomarker, and neuropsychological outcomes. MRI images from the OASIS dataset (19, 20) are analyzed using voxel-based morphometry. Table 1 summarizes the recent work on prediction of Alzheimer's disease.

**Table 1:** Summary of recent work related to AD.

**Research study**	**Dataset**	**Models**	**Average classification accuracy**
Khan et al. (2)	Inage modality	Machine learning and deep learning models	Study on different ML and DL approaches, and different databases related to brain disease
Saratxaga et al. (21)	OASIS dataset	Deep learning and image processing technique	88%
Sudharsan and Thailambal (22)	ADNI dataset	Machine learning models	75%
Helaly et al. (5)	ADNI dataset	Convolutional neural networks	93% for multiclass AD stages
Shakila Basheer et al. (23)	OASIS dataset	Deep neural networks	92%
Martinez-Murcia et al. (3)	ADNI dataset	Deep learning using convolutional autoencoders	80%
Prajapati et al. (4)	ADNI dataset	Deep neural network binary classifier	85%

## Materials and Methods

The proposed approach consists of three basic steps. Firstly, the Alzheimer's disease dataset (24–26) was loaded into pandas for data preprocessing. This study utilized a longitudinal dataset, so a timeline of the study was necessary to gain further insight into the data. Our first step was to determine how cross-sectional the data appear to be, if either at a baseline or at a particular time. A complete analysis of the data was then conducted, including a comparison of the main study parts and the corresponding data collected during each visit. In this work, longitudinal MRI data is our primary data source. MRI data from 150 patients aged from 60 to 96 were included in the study. We scanned each patient at least once. Everyone is right-handed. Throughout the study, 72 of the patients were classified as “non-demented”. At the time of their initial visits, 64 patients were classified as being “Demented,” and they remained in this category throughout the study. Table 2 shows the dataset description of MRI data.

**Table 2:** Dataset description.

**S.No**.	**Attributes**	**Description**
1	ID	Identification
2	M/F	Gender (M if Male, F if Female)
3	Hand	Handedness
4	Age	Age in years
5	EDUC	Years of education
6	SES	Socio Economic Status
7	MMSE	Mini Mental State Examination
8	CDR	Clinical Dementia Rating
9	eTIV	Estimated Total Intracranial Volume
10	nWBV	Normalize Whole Brain Volume
11	ASF	Atlas Scaling Factor
12	Delay	Delay

**Figure 1:**
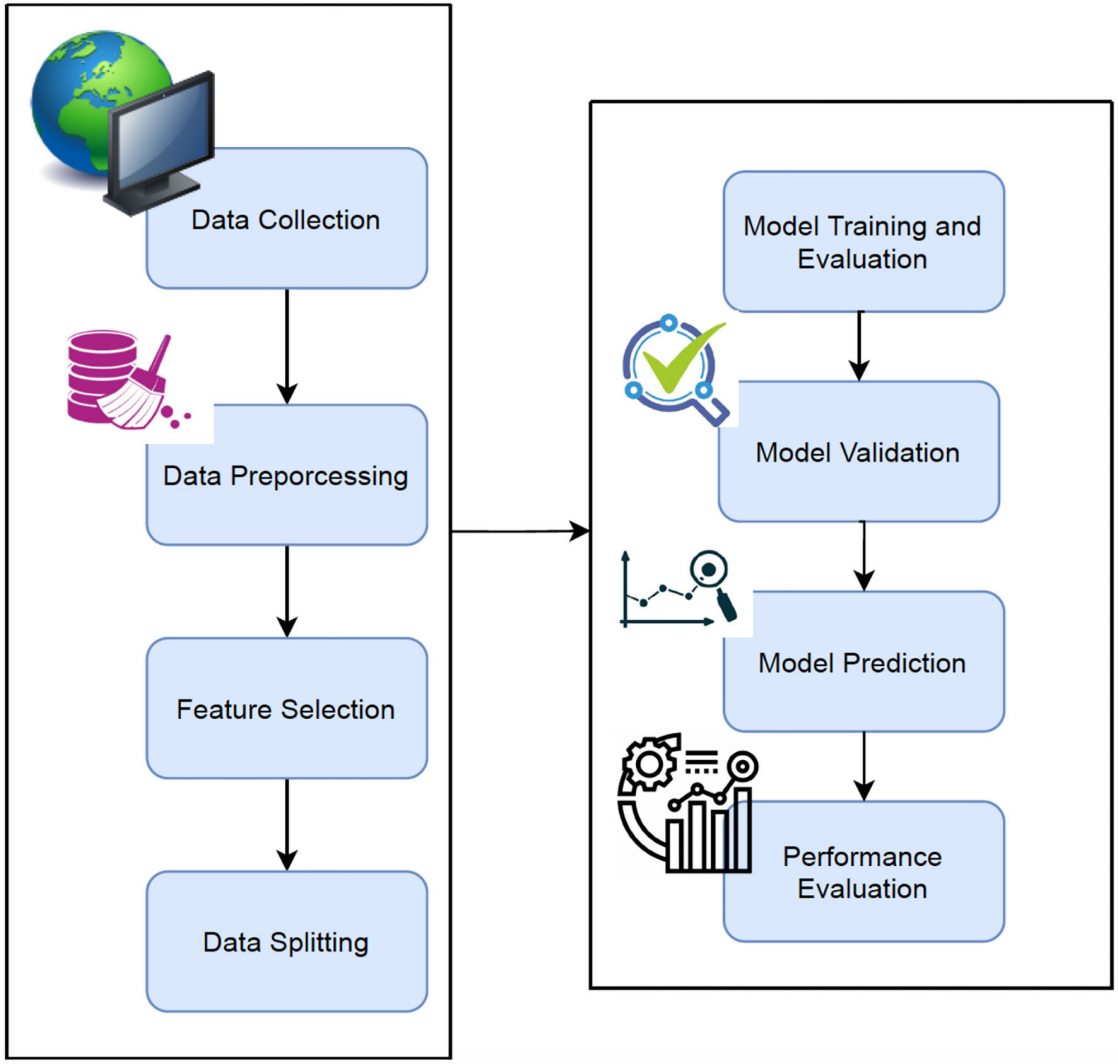
Proposed workflow.

The Machine Learning techniques (26, 27) were applied to Alzheimer's disease datasets to bring a new dimension to predict Disease at an early stage. The raw Alzheimer's disease datasets are inconsistent and redundant, which affects the accuracy of algorithms (28, 29). Before evaluating machine-learning algorithms, data must be effectively prepared for analysis by removing unwanted attributes, missing values, and redundant records. Building a machine-learning model requires splitting the data into training and testing sets. In the following data preparation step, the training data were used to create a model, which was then applied to test data to predict Alzheimer's Disease (28, 30, 31). The model was trained from training set data, and test set data were used to test unseen data. Cross-validation was carried out by dividing the dataset into three subsets. Model predictions are made using one subset of the data (test data) and model performance is evaluated using the other subsets (training and validation) of the data. The data had been preprocessed, and we randomly divided it into an 80:20 ratio, with 80% going to training and 20% gone to testing. Figure 1 describes the system workflow for predicting the Alzheimer's Disease at early stage (32, 33).

### Data Preparation

Various data-mining techniques were used to clean and preprocess the data in this phase. As part of this, missing values are handled, features are extracted, and features are transformed, and so on. In the SES column, we identified 9 rows with missing values (34, 35). This issue is addressed in two ways. The simplest solution is to drop the rows with missing values. The other way to fill in missing values is by Imputation (21), which refers to replacing them with their corresponding values. The model should perform better if we impute since we only have 140 measurements. The 9 rows with missing values are removed in the SES attribute and the median value is used for the imputation.

### Data Analysis

We have discussed the relationships between each feature of an MRI test and dementia in this section. In order to formulate the relationship of data explicitly using a graph, we conducted this Exploratory Data Analysis process (36, 37) to estimate the correlations before extracting data or analyzing it. The information could be used to interpret the nature of the data later on and to determine what method to use to analyze it. Table 3 shows the Min, Max and median values of each attribute.

**Table 3:** Min, max, and median values of each attribute.

**Min**	**Max**	**Mean**	**Median**
EDUC	7	22	14.2
SES	2	6	2.3
MMSE	16	30	26.2
CDR	0	1	0.3
eTIV	1,120	1,990	1,450
nWBV	0.55	0.81	0.7
ASF	0.87	1.43	1.3

### Feature Selection

Feature selection is very important in machine learning. In this work, feature selection is applied to the clinical data of Alzheimer's disease where we have thousands of samples. Feature selection (22) has three methods such as: Filter methods, Wrapper methods, and embedded methods. Filter method is a common method used in the stage of pre-processing. Wrapper methods is another method which core the feature subset. Finally, Embedded method combines the filter and wrapper methods.

The most common and popular feature selection methods are chosen in this work are Correlation coefficient, Information gain, and Chi-Square.

### Correlation Coefficient

The covariance between the two variables X and Y is defined as


$$\begin{array}{l} \rho_{\text{X},\text{Y}}=\;\frac{Cov\left(X,Y\right)}{\sigma_{X}\sigma_{Y}} \end{array}$$

The covariance between two variables measures the linear relationship between them. Using correlation coefficients, it is easy to find a correlation between the various stages of Alzheimer's. The problem with this method is that the data is collected from a broad range of sources, so it becomes very sensitive to outliers.

### Information Gain

The entropy of the lower node is subtracted from the entropy of upper node to obtain the Information gain value when the attribute D is selected.


$$\begin{array}{l} Gain\left(D\right)\;=\;I\left(s1,s2,s3,\ldots \ldots .sn\right)-E\;\left(Feature\;D\right)\; \end{array}$$

**Chi-Square:** Using this method, we can examine categorical variables such as the relationship between food and obesity.


$$\begin{array}{l} Chi-Square\;=\;\frac{{\left(Observed-Expected\right)}^{2}}{expected}\; \end{array}$$

### Preparation and Splitting the Data

**Figure 2:**
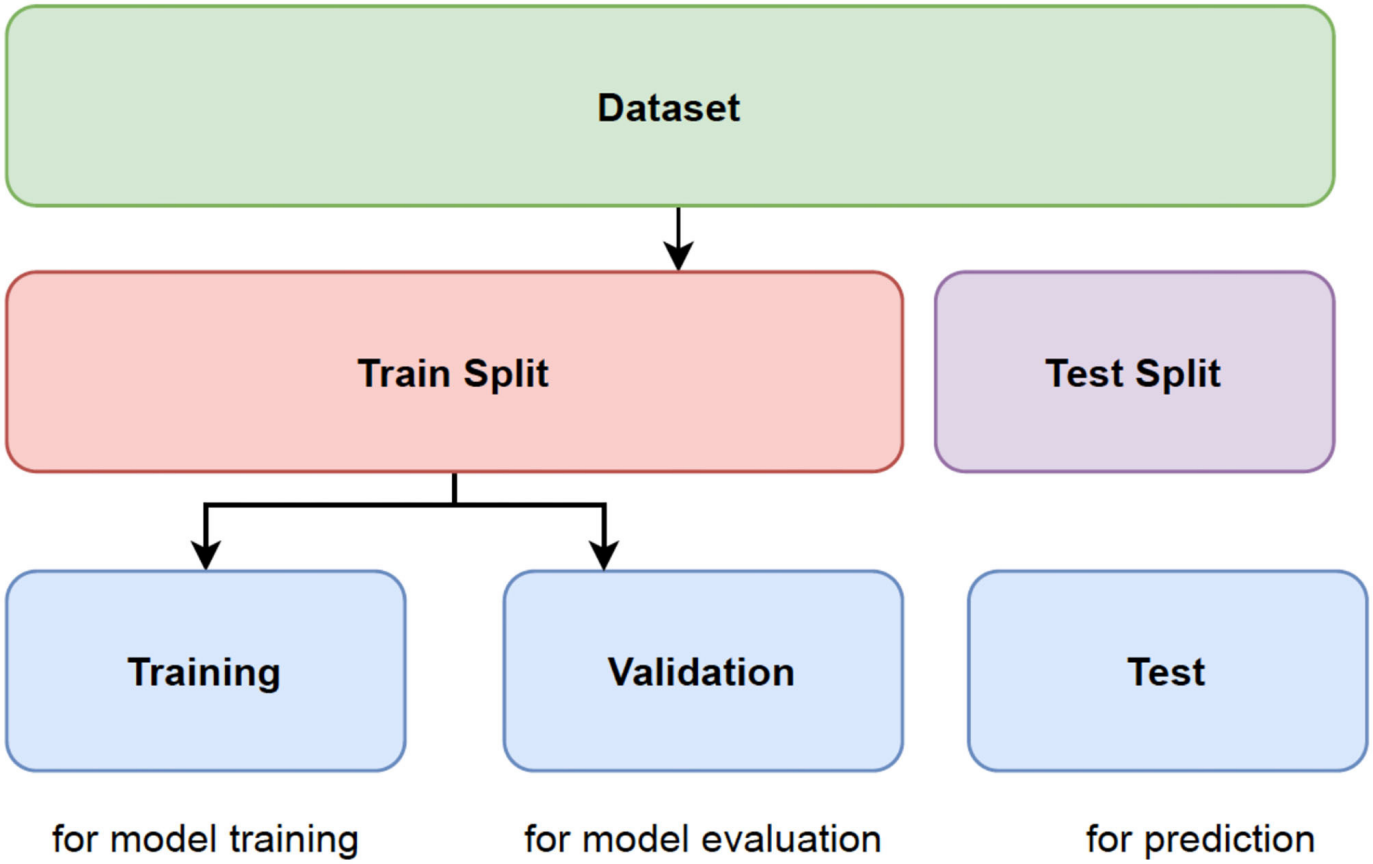
Representation of data splitting.

Figure 2 shows the schematic representation of data splitting stage

1. Select Data: M.F, Age, EDUC, SES, MMSE, eTIV, nWBV, ASF, CDR
2. Train_Data < - round(0.8 ^*^ nrow(data)) #Select 80% of train data
3. TrainData_indices < - sample(1:nrow(data), Train_Data). #Vector is created with random indeces
4. TrainML < - data[TrainData_indices, ] #Training dataset is generated
5. SplitFormula < - CDR ~ M.F + Age + EDUC + SES + MMSE + eTIV + nWBV
6. N = 5
7. Split < - nWayCrossValidation(nrow(data), n). #5-fold cross validation is generated

### Classifier Models

#### Decision Tree (DT)

An overview of the decision tree gives a tree-based model for dividing the data repeatedly based on the cutoff values of the features. Splitting creates subsets by separating instances into subsets. Intermediate subsets are referred to as internal nodes, while leaves are referred to as leaf nodes. A decision tree is most useful when there is significant interaction between the features and the target.

#### Random Forest (RF)

A random forest model performs better than a decision tree because it avoids the problem of overfitting. Models based on random forests consist of various decision trees, each slightly different from the others. Using the majority voting algorithm, the ensemble makes predictions based on each individual decision tree model (bagging). As a result, the amount of overfitting is reduced while maintaining the predictive ability of each tree.

#### Support Vector Machine (SVM)

This method involves determining the class of data points by appropriate hyper planes in a multidimensional space. By using SVM (25), we aim to find a hyperplane that separates cases of two categories of variables that take up neighboring clusters of vectors, one on one side, the other on the other side. Support vectors are those that are closer to the hyperplane. Training and test data are used in SVM. Training data is broken up into target values and attributes. SVM produces a model for predicting target values for test data.

#### XGBoost

XGBoost stands for eXtreme Gradient BOOSTting. It refers to the process of implementing gradient-boosted decision trees for maximum speed and performance. Due to the sequential nature of model training, gradient boosting machines are generally slow in implementation and not very scalable. XGBoost is focused on speed and performance.

#### Voting

Voting is one of the simplest ways of combining the predictions from multiple earning algorithms. Voting classifiers aren't actually classifiers but are more like wrappers for multiple ones that are trained and evaluated concurrently in order to benefit from their specific characteristics. We can train data sets using different algorithms and ensembles then to predict the final output. There are two ways to reach a majority vote on a prediction:

Hard voting: The simplest form of majority voting is hard voting. The class with the most votes (Nc) will be chosen in this case. Our prediction is based on the majority vote of each classifier.

Soft voting: This involves adding up the probability vectors for each predicted class (for all classifiers) and choosing the one that represents the highest value (recommended only when the classifiers are well calibrated).

### Model Validation

Model validation reduces the overfitting problem. Cross Validation is done to train the ML model and are used to calculate the accuracy of the model. It is a challenging task to make the ML model from noise free. Hence, in this research work, Cross validation is performed which divides the whole dataset into n divisions which is of equal in size. The ML model is trained for every iteration with the n-1 divisions. The performance of the method is analyzed by the mean of all *n*-folds. In this work, the ML model was trained and tested 10 times by applying ten-fold cross validation to the model.

## Results and Discussions

We evaluate various performance metrics like accuracy, precision, recall and F1 score. To determine the best parameters for each model, we perform 5-fold cross-validation: Decision Tree, SVM, Random Forests, XGBoost and Voting. Finally, we compare accuracy of each model. Several metrics and techniques were used to identify overfitting and parameter tuning issues after the models were developed. Performance evaluations can either be binary or multiclass and are described using the confusion matrix. A learning model was developed to distinguish true Alzheimer's disease affected people from a given population and a novel Machine Learning classifier was developed and validated to predict and separate true Alzheimer's disease affected people. The following evaluation measures were calculated using these components: precision, recall, accuracy, and F-score. Based on this study, recall (sensitivity) is the proportion of people accurately classified as having Alzheimer's. The precision of Alzheimer's diagnosis is the rate of people correctly classified as not having the disease. Alternatively, F1 represents the weighted average of recall and precision, while accuracy represents the proportion of people correctly classified. According to the results, the patient receives a report that tells him or her what stage of Alzheimer's Disease he or she is currently in. It is very important to detect the stages because the stages are based on the responses of the patients. In addition, knowing the stage helps doctors better understand how the Disease is affecting them. This research used these environments, tools, and libraries to conduct its experiments and analysis:

a) Environments Used: Python 3
b) Scikit-learn libraries for machine learning

**Figure 3:**
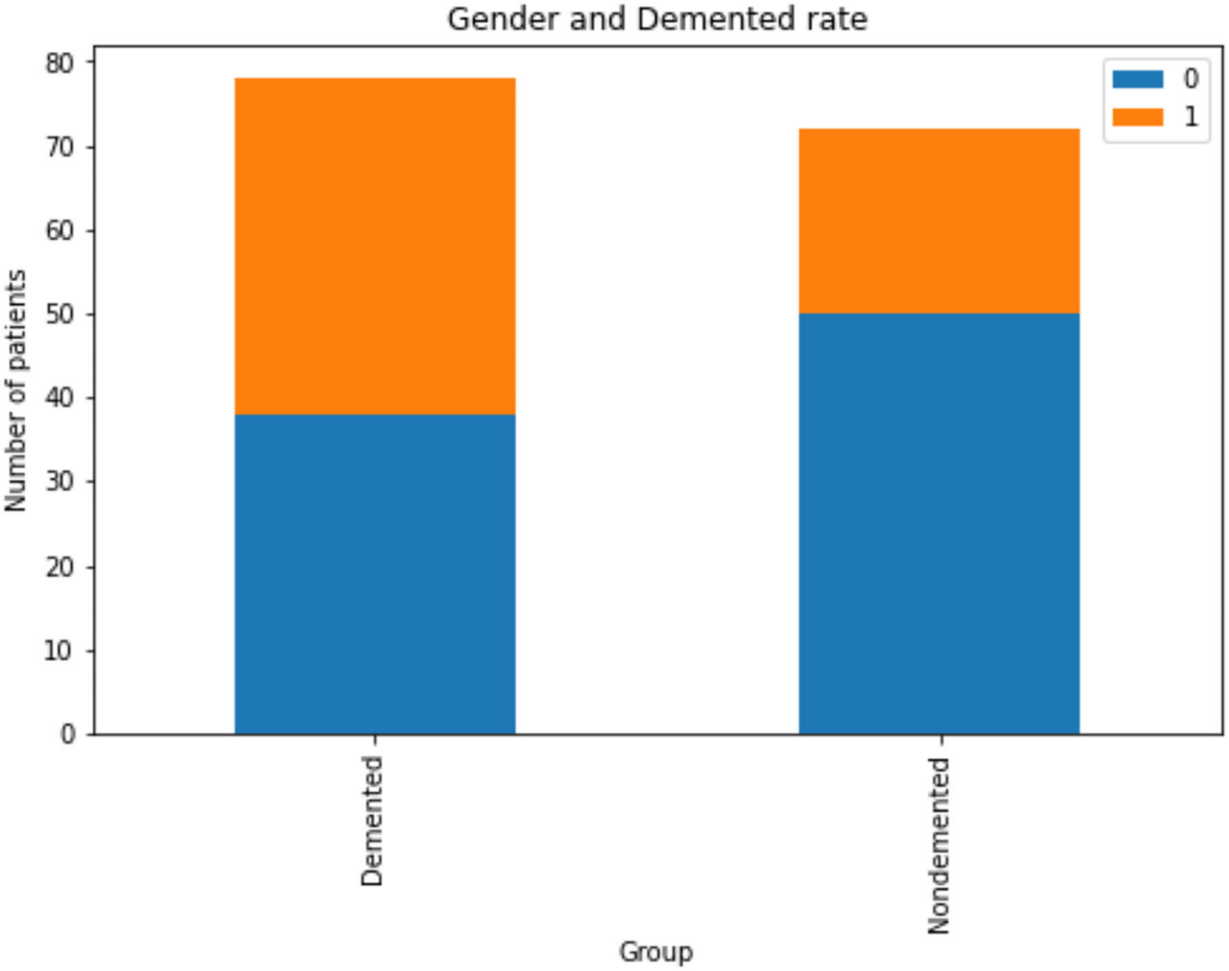
Analysis of demented and non-demented rate based on gender, Gender group Female = 0, Male = 1.

**Figure 4:**
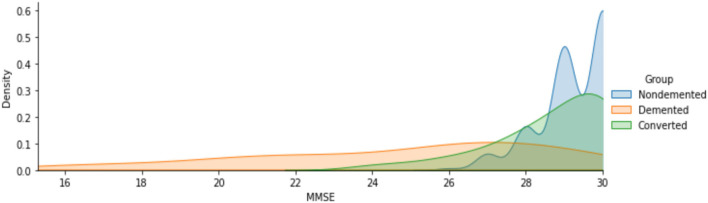
Analysis of MMSE scores for demented and non-demented group of patients.

The Figure 3 indicates that men are more likely than women to have dementia. Figure 4 that the non-demented group had much higher MMSE (Mini-Mental State Examination) scores than those with dementia.

**Figure 5:**
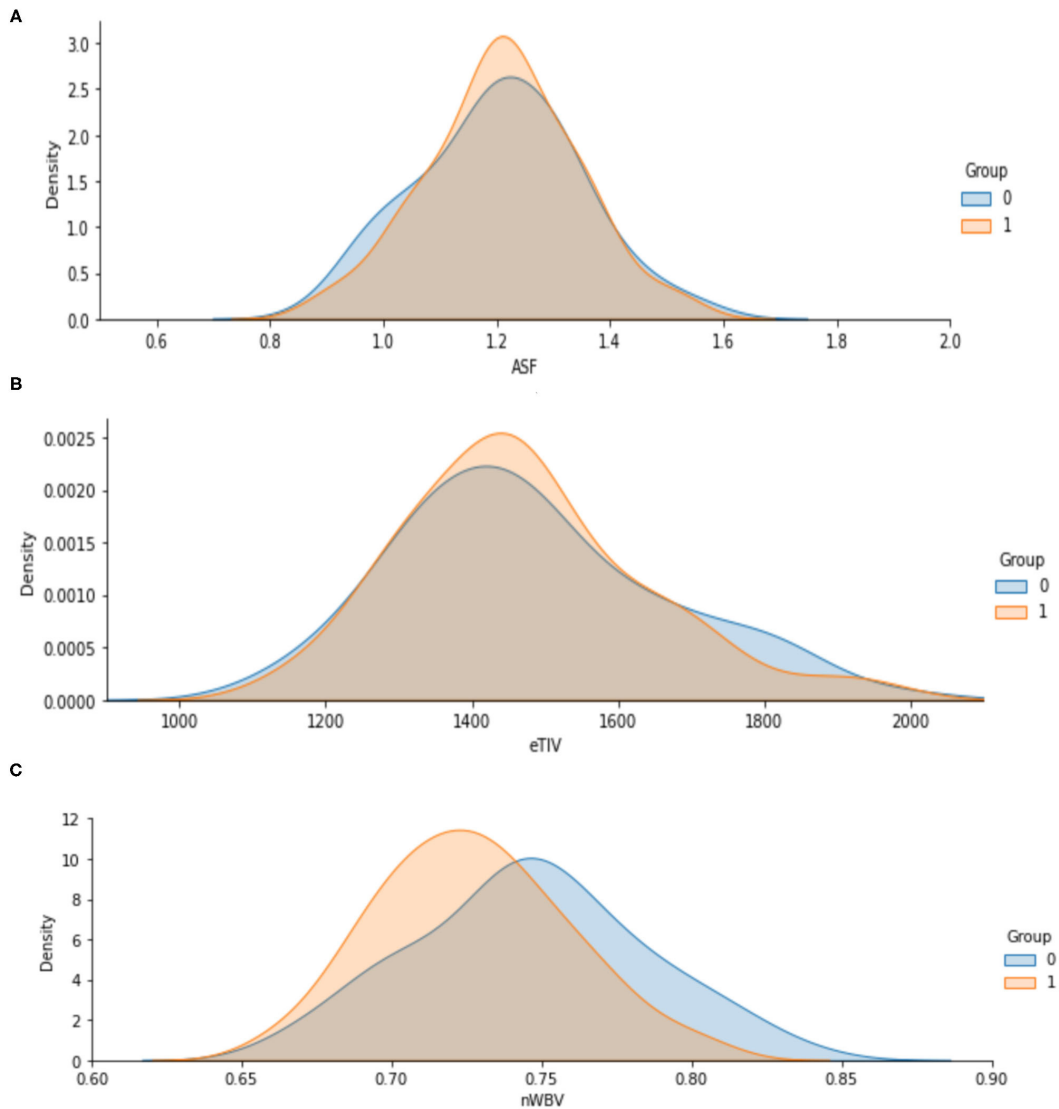
**(A–C)** Analysis of ASF, eTIV and nWBV for Demented and Non-demented group.

**Figure 6:**
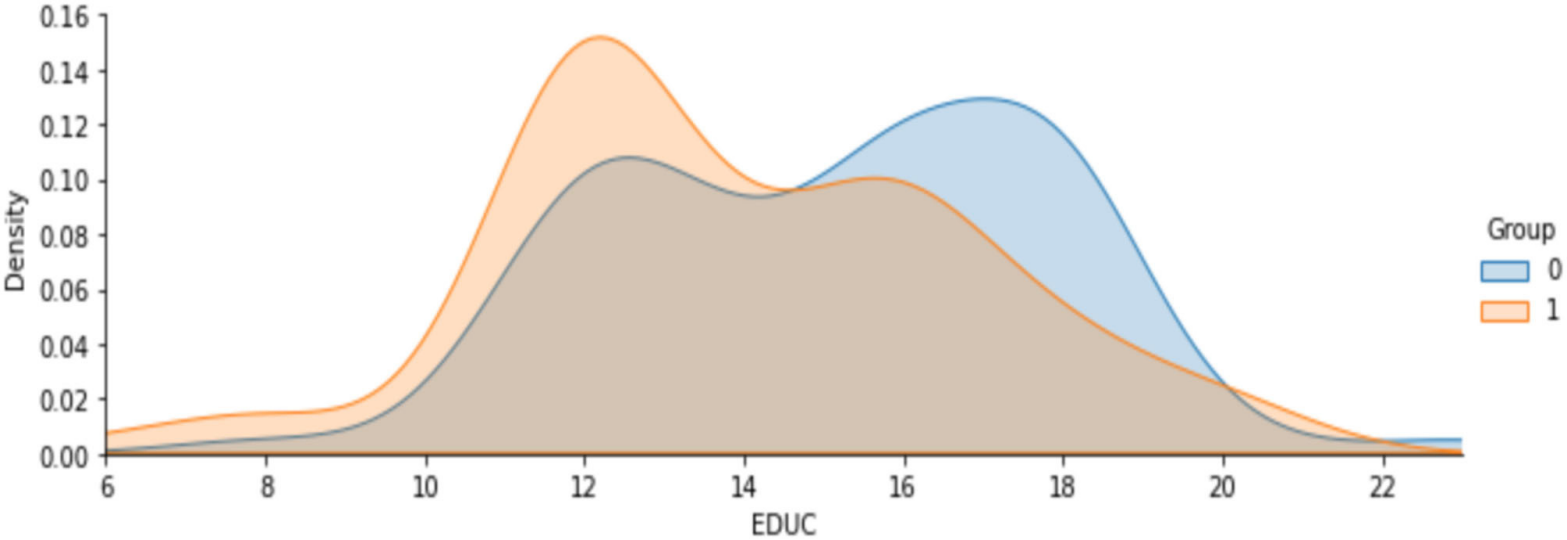
Analysis on years of education.

The Figures 5A–C shows the analyzed value of ASF, eTIV and nWBV for Demented and Non-demented group of people. As indicated by the graph in Figure 5, the Non-demented group has a higher brain volume ratio than the Demented group. The reason for this is that the diseases influence the brain tissues causing them to shrink. Figure 6 shows the analyzed results of EDUC for Demented and Non-demented people.

**Figure 7:**
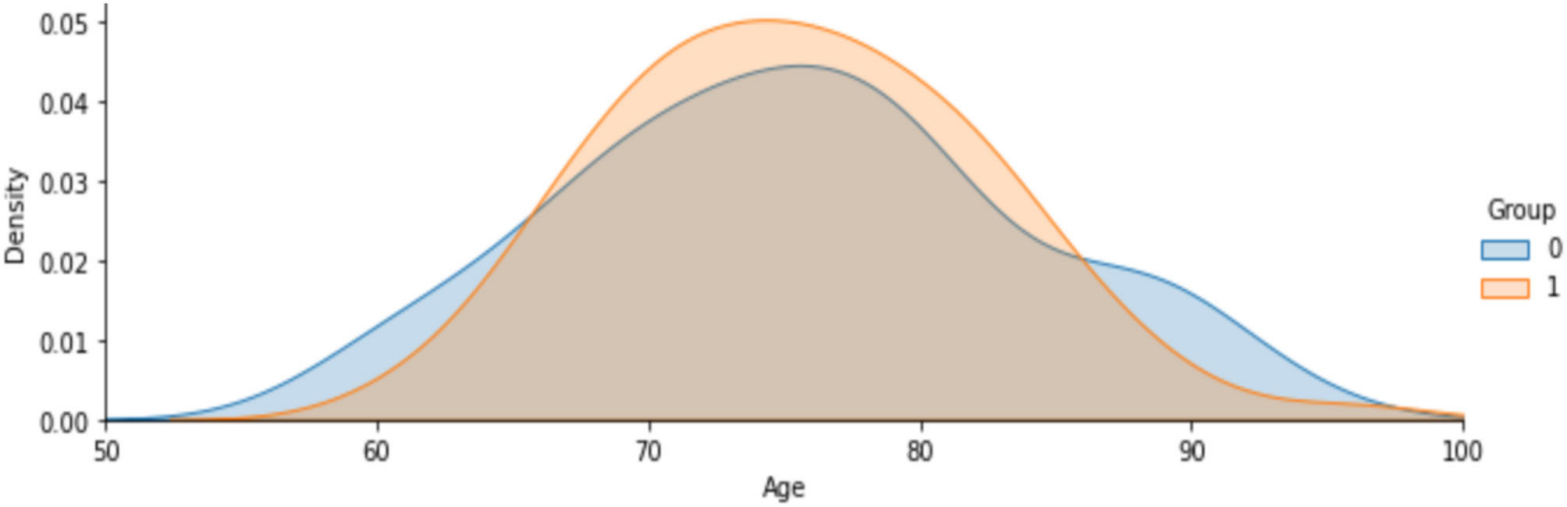
Analysis on people affected by demented and non-demented group based on age.

Figure 7 shows the analysis on age attribute to find the percentage of people affected based on the demented and non-demented group. It is observed that a higher percentage of Demented patients are 70-80 years old than non-demented patients. It is likely that people with that kind of Disease have a low survival rate. Only a few people are over 90 years old.

From the above all analysis on the attributes, the following are the summary on intermediate results.

1. It is more likely for men to have demented, or Alzheimer's Disease, than for women.
2. In terms of years of education, demented patients were less educated.
3. Brain volume in non-demented groups is greater than in demented groups.
4. Among the demented group there is a higher concentration of 70-80-year-olds than in the non-demented patients.

Table 4 shows the performance comparison of accuracy, precision, recall, and F1 score for different ML models. The performance measures are defined as,

**Table 4:** Performance comparison of different ML models.

**Model**	**Accuracy**	**Precision**	**Recall**	**F1-score**
Decision tree classifier	80.46%	0.80	0.79	0.78
Random forest classifier	86.92%	0.85	0.81	0.80
Support vector machine	81.67%	0.77	0.70	0.79
XGBoost	85.92%	0.85	0.83	0.85
Voting classifier	85.12%	0.83	0.83	0.85

**Accuracy:** It is the measure of finding the proportion of correctly classified result from the total instances.


$$\begin{array}{l} Accuracy\left(inPercentage\right)=\frac{TN+TN}{TP+TN+FP+FN}\times 100 \end{array}$$

**Precision:** This measures the number of correctly predicted positive rate divided by the total predicted positive rates. If the Precision value is 1, it is meant as a good classifier.


$$\begin{array}{l} Precision=\frac{TP}{TP+FP} \end{array}$$

**Recall:** Recall is a true positive rate. If the recall is 1, it is meant as a good classifier.


$$\begin{array}{l} Recall\left(inPercentage\right)=\frac{TP}{TP+FN} \end{array}$$

**F1 Score:** It is a measure which considers both Recall and Precision parameters. F1 score becomes 1 only when both the measure such as Recall and Precision is 1.


$$\begin{array}{l} F1Score\left(inPercentage\right)=2^{*}\frac{Recall^{*}Precision}{Recall+Precision} \end{array}$$

**Figure 8:**
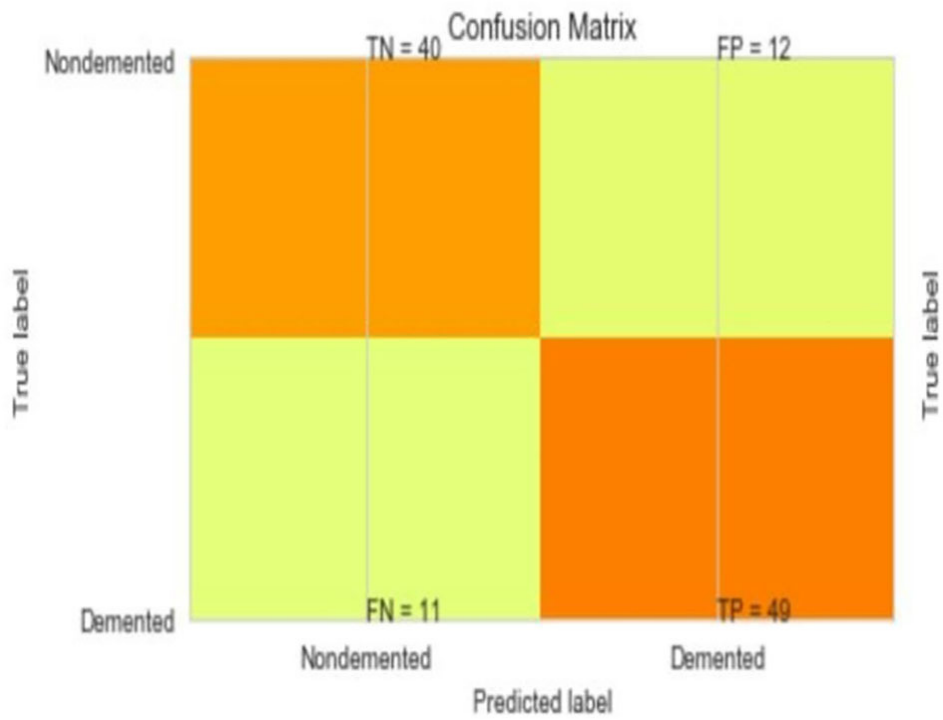
Confusion matrix for decision tree.

**Figure 9:**
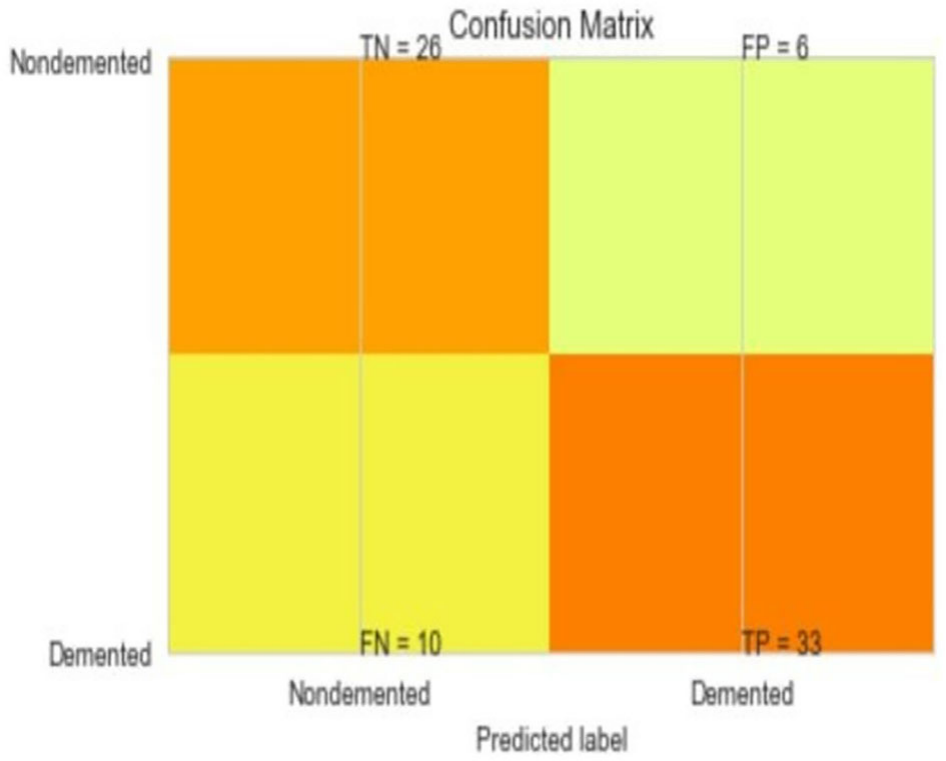
Confusion matrix for random forest.

**Figure 10:**
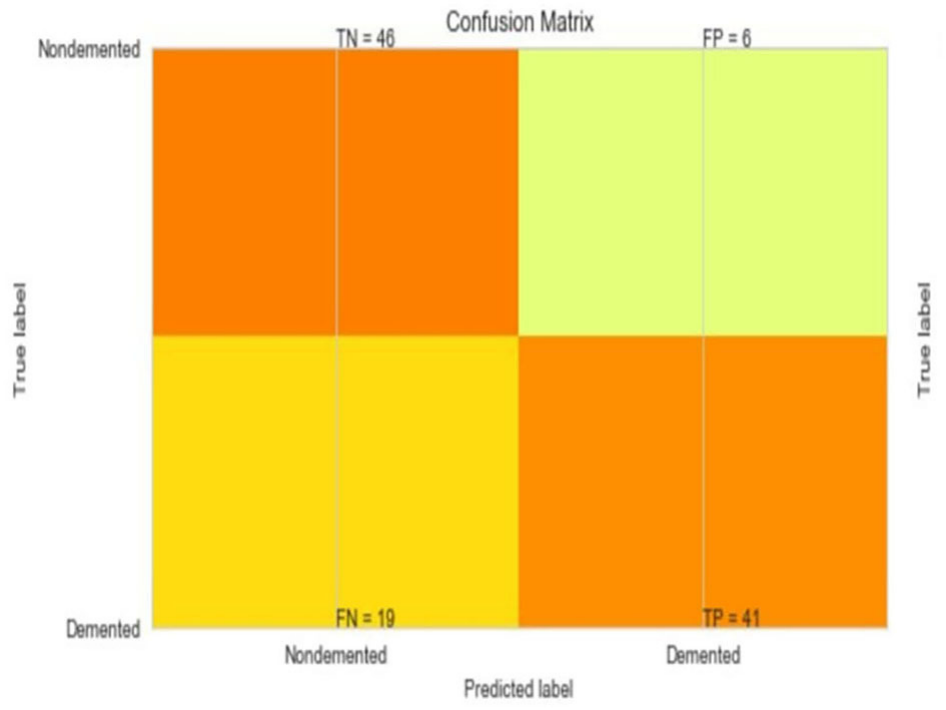
Confusion matrix for SVM.

**Figure 11:**
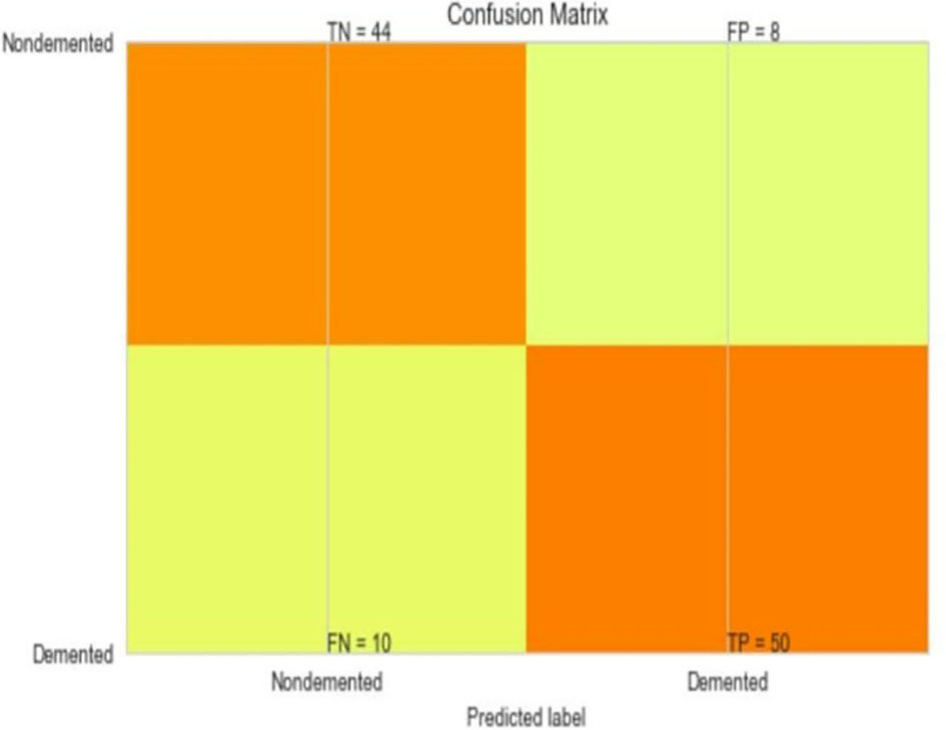
Confusion matrix for XGBoost.

**Figure 12:**
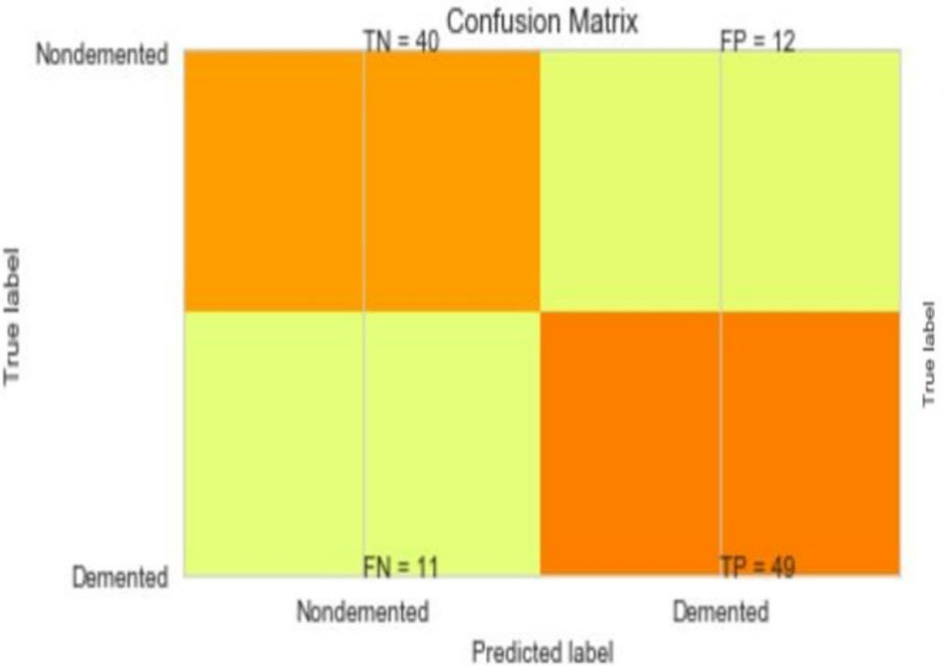
Confusion matrix for soft voting classifier.

**Figure 13:**
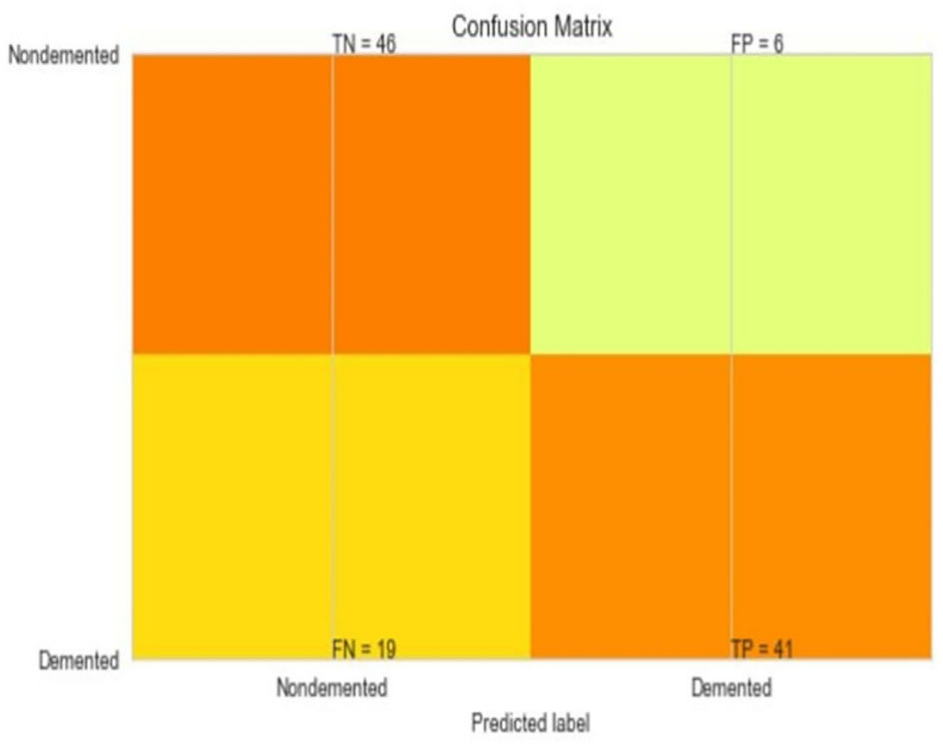
Confusion matrix for hard voting classifier.

The most common metrics are the conversions of the True Positive (TP), the False Positive (FP), the True Negative (TN), and the False Negative (FN) metrics. Figures 8–13 shows the confusion matrix for Decision tree, Random Forest, SVM, XG boost, Soft, and Hard Voting classifier ML models.

**Figure 14:**
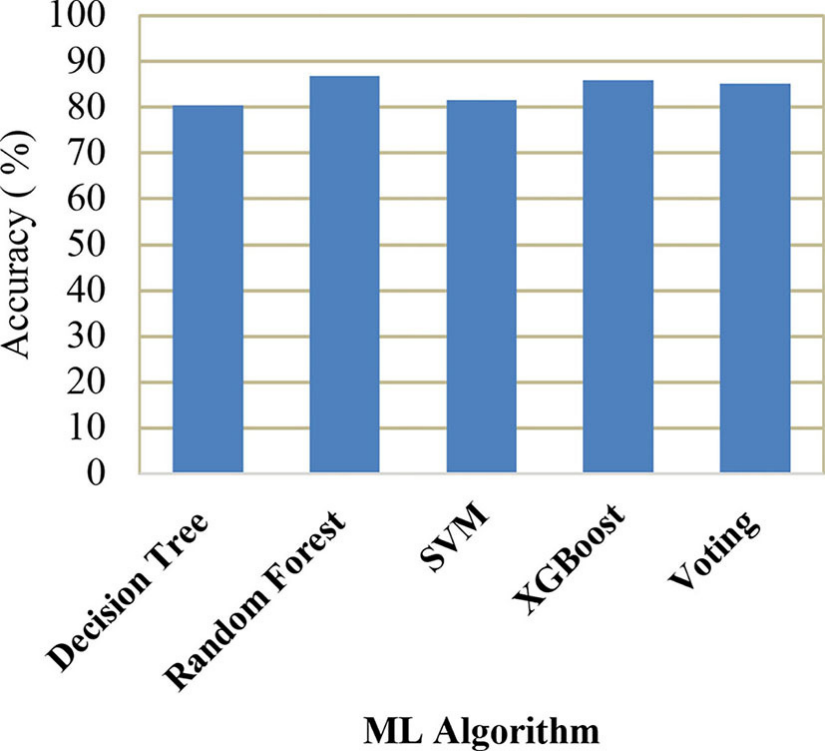
Comparison of accuracy.

**Figure 15:**
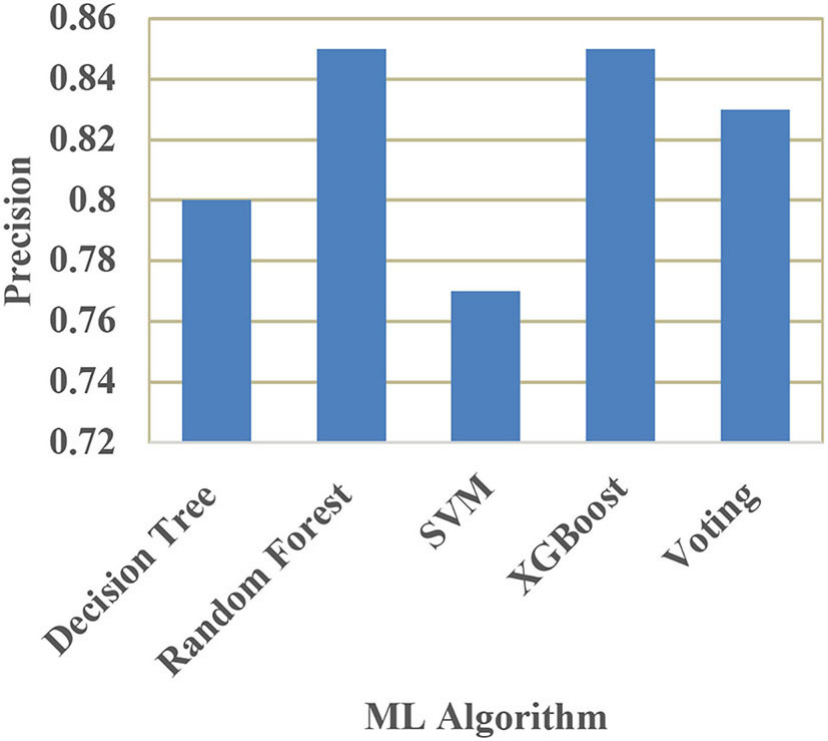
Comparison of precision.

**Figure 16:**
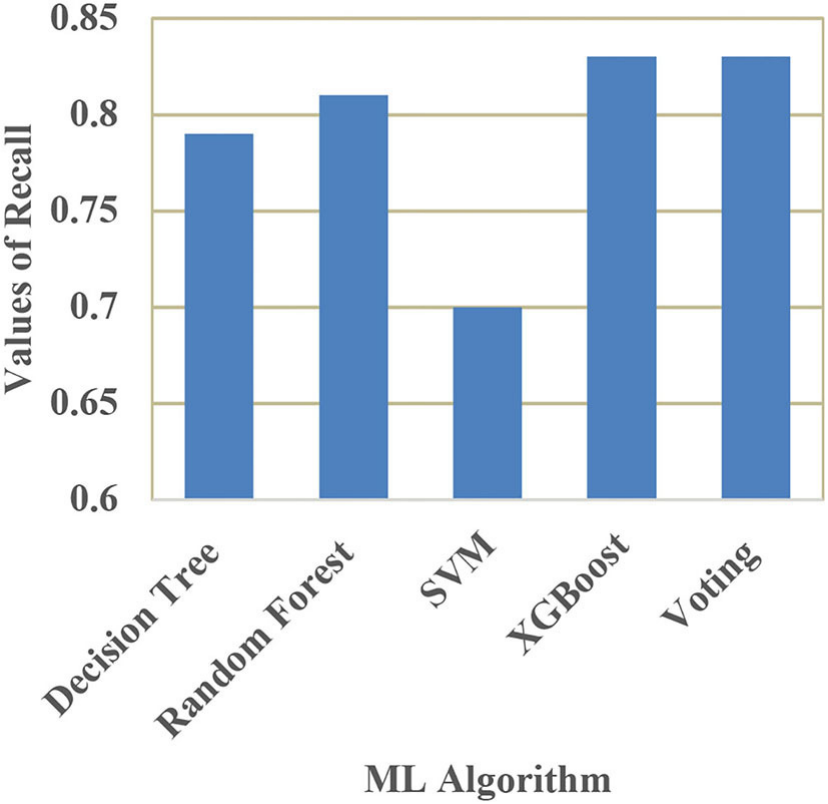
Comparison of recall.

**Figure 17:**
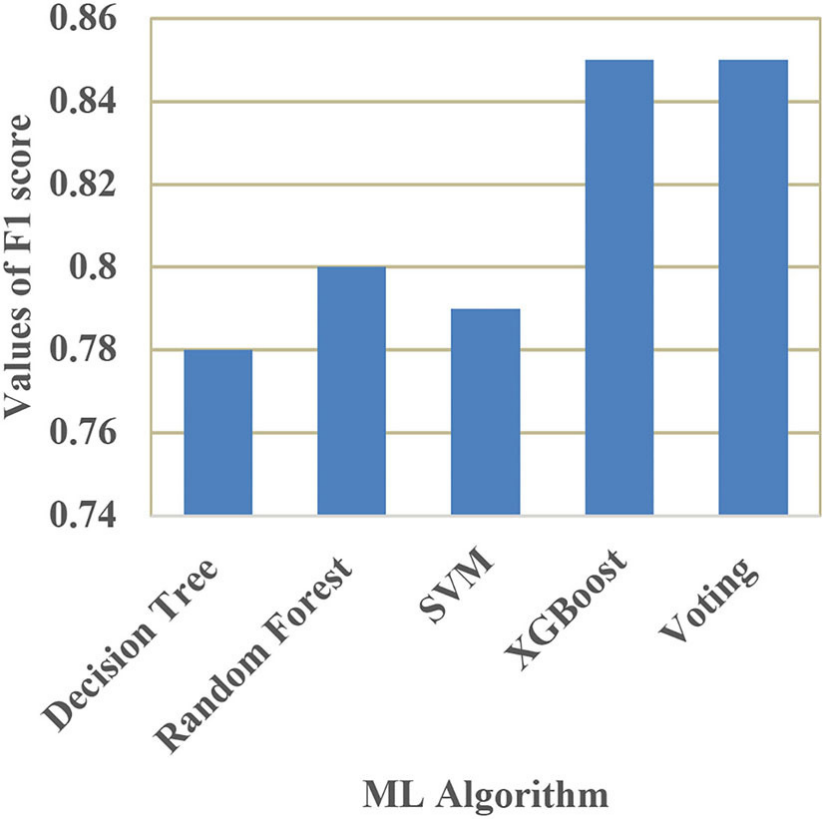
Comparison of F1 score.

A comparison of training and testing accuracy has been conducted for each model to eliminate overfitting. For each model, precision, recall, accuracy, and F1-score are shown in Table 3. Based on the analysis showed in the Table 3, the results approved that the best and ideal techniques, which have a good performance, are random forest, and XGBoost. The accuracy value of Voting classifier model is also closer to the random forest, and XGBoost models. All the experimental results (the average accuracy, precision, recall, and F measure of each model) were collected for extra analysis. The comparative analyses among all the Machine Learning models in terms of accuracy, precision, recall, and F1 score are presented graphically in Figures 14–17 respectively.

## Conclusions

Alzheimer's is a major health concern, and rather than offering a cure, it is more important to reduce risk, provide early intervention, and diagnose symptoms early and accurately. As seen in the literature survey there have been a lot of efforts made to detect Alzheimer's Disease with different machine learning algorithms and micro-simulation methods; however, it remains a challenging task to identify relevant attributes that can detect Alzheimer's very early. The future work will focus on the extraction and analysis of new features that will be more likely to aid in the detection of Alzheimer's Disease, and on eliminating redundant and irrelevant features from existing feature sets to improve the accuracy of detection techniques. By adding metrics like MMSE and Education to our model, we'll be able to train it to distinguish between healthy adults and those with Alzheimer's.

## Data Availability Statement

Publicly available datasets were analyzed in this study. This data can be found at: https://www.kaggle.com/jboysen/mri-and-alzheimers?select=oasis_cross-sectional.csv.

## Author Contributions

CK: research concept and methodology and writing—original draft preparation. VM: review and editing. SS: supervision. OK and CT: validation. All authors contributed to the article and approved the submitted version.

## Funding

This research has been funded by Dirección General de Investigaciones of Universidad Santiago de Cali under call No. 01-2021.

## Conflict of Interest

The authors declare that the research was conducted in the absence of any commercial or financial relationships that could be construed as a potential conflict of interest.

## Publisher's Note

All claims expressed in this article are solely those of the authors and do not necessarily represent those of their affiliated organizations, or those of the publisher, the editors and the reviewers. Any product that may be evaluated in this article, or claim that may be made by its manufacturer, is not guaranteed or endorsed by the publisher.

## References

[1] Sivakani GA Ansari R. Machine learning framework for implementing Alzheimer's disease. Int Conferen Commun Signal Process. (2020) 12:588–92. 10.1109/ICCSP48568.2020.918222027295638

[2] Khan P Kader MF Islam SR Rahman AB Kamal MS Toha MU. Machine learning and deep learning approaches for brain disease diagnosis: principles and recent advances. IEEE Access. (2021) 9:37622–55. 10.1109/ACCESS.2021.3062484

[3] Martinez-Murcia FJ Ortiz A Gorriz JM Ramirez J Castillo-Barnes D. Studying the manifold structure of Alzheimer's disease: a deep learning approach using convolutional autoencoders. IEEE J Biomed Health Inform. (2020) 24:17–26. 10.1109/JBHI.2019.2914970 31217131

[4] Prajapati R Khatri U Kwon GR. “An efficient deep neural network binary classifier for alzheimer's disease classification,” In: International Conference on Artificial Intelligence in Information and Communication (ICAIIC). (2021), p. 231–234.

[5] Helaly HA Badawy M Haikal AY. Deep learning approach for early detection of Alzheimer's disease. Cogn Computing. (2021) 21:1–17. 10.1007/s12559-021-09946-2 34745371 PMC8563360

[6] Yaffe K. Modifiable risk factors and prevention of dementia: what is the latest evidence. JAMA Intern Med. (2018) 178:281–2. 10.1001/jamainternmed.2017.7299 29255907

[7] Livingston G Sommerlad A Orgeta V Costafreda SG Huntley D. Dementia prevention, intervention, and care. The Lancet. (2017) 390:2673–73. 10.1016/S0140-6736<17>31363-6 28735855

[8] O'Donnell CA Manera V Köhler S Irving K. Promoting modifiable risk factors for dementia: is there a role for general practice? British J General Pract. (2015) 65:567–8. 10.3399/bjgp15X687241 26500299 PMC4617245

[9] Sulaiman N Abdulsahib G Khalaf O Mohammed MN. “Effect of Using Different Propagations of OLSR and DSDV Routing Protocols”, In Proceedings of the IEEE International Conference on Intelligent Systems Structureing and Simulation. (2014), pp. 540–5.

[10] Deckers K van Boxtel MP Schiepers OJ de Vugt M Muñoz Sánchez JL Anstey KJ. Target risk factors for dementia prevention: a systematic review and Delphi consensus study on the evidence from observational studies. Int J Geriatric Psychiatry. (2015) 30:234–46. 10.1002/gps.4245 25504093

[11] Schiepers OJ Köhler S Deckers K Irving K O'donnell CA Van den Akker. Lifestyle for Brain Health (LIBRA): a new model for dementia prevention. Int J Geriatric Psychiatry. (2018) 33:167–75. 10.1002/gps.4700 28247500

[12] Vos SJ Van Boxtel MP Schiepers OJ Deckers K De Vugt M Carrière I. Modifiable risk factors for prevention of dementia in midlife, late life and the oldest-old: validation of the LIBRA Index. J Alzheimer's Dis. (2017) 58:537–47. 10.3233/JAD-161208 28453475

[13] Osamh Khalaf I Ghaida M Abdulsahib D. Energy efficient routing and reliable data transmission protocol in WSN. Int J Adv Soft Comput Applicat. (2020) 12:45–53.

[14] National National Academies of Sciences Engineering and Medicine. Preventing cognitive decline and dementia: A way forward. London: The National Academies Press (2018). 28650595

[15] Tariq S Barber PA. Dementia risk and prevention by targeting modifiable vascular risk factors. J Neurochemistr. (2018) 144:565–81. 10.1111/jnc.14132 28734089

[16] Williams Jennifer A Weakley A Cook MS Edgecombe DJ. “Machine learning techniques for diagnostic differentiation of mild cognitive impairment and dementia,” In Workshops at the Twenty-Seventh AAAI Conference on Artificial Intelligence. (2018), pp. 71–6.

[17] Khalaf OI Sabbar BM. A modified algorithm for improving lifetime WSN. J Eng Appl Sci. (2018) 13:9277–82.

[18] Khalaf OI Abdulsahib GM Sabbar BM. Optimization of wireless sensor network coverage using the Bee Algorithm. J Inf Sci Eng. (2020) 36:377–86.22163942

[19] Chi CL Oh Borson WS. “Feasibility Study of a Machine Learning Approach to Predict Dementia Progression,” in International Conference: In Health care Informatics (ICHI). (2015), p. 450.

[20] Chyzhyk A Savio D. Feature extraction from structural MRI images based on VBM: data from OASIS database, University of The Basque Country, Internal research publication (2010).

[21] Saratxaga CL Moya I Picón A Acosta M Moreno-Fernandez-de-Leceta A Garrote E. MRIDeep learning-based solution forAlzheimer's Disease Prediction. J.Pers. Med. (2021) 11:902. 10.3390/jpm11090902 34575679 PMC8466762

[22] Sudharsan M Thailambal G. Alzheimer's disease prediction using machine learning techniques and principal component analysis (PCA), Materials Today: Proceedings (2021).

[23] Basheer S Bhatia S Sakri SB. “Computational Modeling of Dementia Prediction Using Deep Neural Network: Analysis on OASIS Dataset,” in IEEE Access. (2021) 9:42449–42462. 10.1109/ACCESS.2021.3066213

[24] Khalaf OI Abdulsahib GM. Frequency estimation by the method of minimum mean squared error and P-value distributed in the wireless sensor network. J Informat Sci Eng. (2019) 35:1099–112.

[25] Ogudo KA Muwawa J Ibrahim Khalaf O Daei Kasmaei H. A device performance and data analytics concept for smartphones' IoT services and machine-type communication in cellular networks. Symmetry. (2019) 11:593–609. 10.3390/sym11040593

[26] Reddy GT Reddy MP Lakshmanna K Rajput DS Kaluri R Srivastava G. Hybrid genetic algorithm and a fuzzy logic classifier for heart disease diagnosis. Evol Intel. (2020) 13:185–96. 10.1007/s12065-019-00327-1

[27] Abdulsahib GM Khalaf OI. Accurate and effective data collection with minimum energy path selection in wireless sensor networks using mobile sinks. J Informat Technol Manage. (2020) 13:139–53.

[28] Gadekallu TR Khare N Bhattacharya S Singh S Maddikunta PK Srivastava G. Deep neural networks to predict diabetic retinopathy. J Ambient Intell Human Computing. (2020) 21:1–4. 10.1007/s12652-020-01963-7

[29] Salman AD Khalaf OI Abdulsahib GM. An adaptive intelligent alarm system for wireless sensor network. Indonesian J Electric Eng Comput Sci. (2019) 15:142–7. 10.11591/ijeecs.v15.i1.pp142-147

[30] Srinivasu PN Bhoi AK Jhaveri RH Reddy GT Bilal M. Probabilistic Deep Q Network for real-time path planning in censorious robotic procedures using force sensors. J Real-Time Image Proc. (2021) 18:773–1785. 10.1007/s11554-021-01122-x

[31] Khalaf OI Abdulsahib GM Kasmaei HD Ogudo KA. A new algorithm on application of blockchain technology in live stream video transmissions and telecommunications. Int J e-Collaboration (IJeC). (2020) 16:16–32. 10.4018/IJeC.2020010102

[32] Abdulsahib GM Khalaf OI. An improved algorithm to fire detection in forest by using wireless sensor networks. Int J Civil Eng Technol (IJCIET). (2018) 9:369–77.

[33] Abdulsahib GM Khalaf IO. Comparison and evaluation of cloud processing models in cloud-based networks. Int J Simul Syst Sci Technol. (2018) 19:5. 10.5013/IJSSST.a.19.05.26

[34] Khalaf OI Abdulsahib GM. Optimized dynamic storage of data (ODSD) in IoT based on blockchain for wireless sensor networks. Peer-to-Peer Netw. (2021) 21:255. 10.1007/s12083-021-01115-4

[35] Javed AR Fahad LG Farhan AA Abbas S Srivastava G Parizi RM. Automated cognitive health assessment in smart homes using machine learning. Sustain Cities Soc. (2020) 65:102572. 10.1016/j.scs.2020.102572

[36] Maddikunta PR Gadekallu TR Iwendi C. Identification of malnutrition and prediction of BMI from facial images using real-time image processing and machine learning. IET Image Process. (2021) 21:1–12. 10.1049/ipr2.12222

[37] Javed AR Sarwar MU ur Rehman S Khan HU Al-Otaibi YD Alnumay WS. PP-SPA: privacy preserved smartphone-based personal assistant to improve routine life functioning of cognitive impaired individuals. Neural Process Lett. (2021) 21:1–8. 10.1007/s11063-020-10414-5

